# Investigating the Clinical Effect of Kinesio Tape on Muscle Performance in Healthy Young Soccer Players – A Prospective Cohort Study

**DOI:** 10.6061/clinics/2019/e1158

**Published:** 2019-09-19

**Authors:** Saud M Alrawaili

**Affiliations:** Department of Physical Therapy and Health Rehabilitation, College of Applied Medical Sciences, Prince Sattam Bin Abdulaziz University, Alkharj, Saudi Arabia

**Keywords:** Kinesio Taping, Muscle Performance, Soccer Players

## Abstract

**OBJECTIVE:**

Kinesio tape (KT) is a visible adhesive restorative tape that has typically been utilized for injury prevention, recovery, and even performance improvement, but limited studies have assessed the effect of KT on muscle performance. The purpose of this study was to investigate the clinical impact of KT on muscle performance in healthy young soccer players.

**METHODS:**

Between 25 March and 21 April 2017, sixteen healthy soccer players with a mean age of 20±2.17 were enrolled in this prospective cohort study. All participants were selected from the college football team of Prince Sattam Bin Abdulaziz University. The muscle performance of the players was evaluated with an isokinetic dynamometer for the following three conditions: without tape, immediately after applying KT, and 8 hrs post-KT application while the tape remained on the same site.

**RESULTS:**

The differences in peak torque and total work among the three conditions were nonsignificant (*p*>0.05). Additionally, applying KT to the thigh muscles did not decrease or increase the performance of non injured healthy soccer players (*p*>0.05).

**CONCLUSION:**

KT does not lead to beneficial outcomes of muscle performance in healthy young soccer players.

## INTRODUCTION

Kinesio tape (KT) was created by Kenzo Kase in 1996 and is a type of adherent tape. KT appears as a thin and versatile tape that can extend up to one hundred-twenty to one hundred-forty percent of its original length; KT is highly flexible and thus brings about fewer system limitations than traditional tape. Kinesio taping is system for wrapping KT that was suggested by Kase; KT is believed to have the capacity to lessen swelling, muscle spasms, and pain as well as prevent sports injuries 1.

KT is a commonly found adhesive restorative tape that has typically been utilized for injury prevention, recovery, and even performance improvement. KT has all the earmarks of being medically effective in controlling pain 2. The restorative impacts of KT for the knee include limiting pain, increasing muscle strength, enhancing gait measures and improving the functional results of patients with sports injuries, osteoarthritis, and patellofemoral pain 3,4.

KT may increase or decrease muscle strength, and numerous studies have reported fundamental psychological components, including neurofacilitation and mechanical limitation; Macgregor et al. recognized the connection between cutaneous afferent stimulation and motor unit firing 5. On the other hand, Cools et al. suggested the non-noteworthy impact of KT on the electrical activity of the shoulder muscles in healthy subjects 2. Few investigations have estimated the adequacy of KT, and these examinations have acquired conflicting results 1,6. The fundamental psychological motivation behind this examination is to evaluate whether muscle performance can be affected by KT since a very limited number of studies have examined the effect of KT on muscle performance. Along these lines, the current study aimed to evaluate the clinical impact of KT on muscle performance when utilized on the front of the thigh and knee and the immediate and delayed impacts of KT that might be important for medical applications since there is a theory that KT application has no medical impact on muscle performance in soccer players. In contrast, the current study hypothesized that KT could improve muscle performance in soccer players.

## METHODS

### Ethical approval

All the study procedures that included human participants were in accordance with the ethical standards of the institutional and/or national research committee and with the 1964 Helsinki Declaration and its later amendments or comparable ethical standards.

### Subjects and study design

Between 25 March and 21 April 2017, sixteen healthy male soccer players with a mean age of 20±2.17, height of 170±4.3, and weight of 68±5.14 were enrolled in this prospective cohort study. All participants were chosen from the college football team at Prince Sattam bin Abdulaziz University. Soccer players with active knee pain, injuries or a history of medical surgery to the lower extremities were excluded from this study. The current study was approved by the localized ethical committee of the Physical Therapy and Health Rehabilitation Department (RHPT/2017/005), College of Applied Medical Sciences, Prince Sattam bin Abdulaziz University. All participants signed written informed consent forms for study participation before enrolling in the investigation.

The Cypex isokinetic dynamometer (Cybex Norm, Humac, CA, USA) was adjusted before every data acquisition session and was used to evaluate the strength of the thigh muscles, including the quadriceps and hamstring muscles (concentric and eccentric), during contractions at a rate of 60°/s and 180°/s.

All soccer players had Y-molded KT applied on the quadriceps muscle by a trained and experienced physiotherapist based on the Kenzo Kase's KT manual 7. The predominant sides of the players' knees were taped. The soccer players were assessed while in a bent lying position while flexing their hip (30°) and knee (60°). The KT was connected 10 cm from a point below the foremost prevalent anterior superior iliac spine (ASIS), was divided at the intersection between the patella and quadriceps muscles tendon, surrounded the patella, and ended inferiorly at the tibial tuberosity. The initial 5 cm of the KT was not stretched and was applied as the fixed point. The small piece between the fixed point and suprapatella was extended by more than one hundred-twenty percent. The portion of KT surrounding the patella remained unstretched 8.

The following three states of KT were assessed for each player: without KT (WKT), immediately after KT (IKT), and 8 hrs post-KT (8KT) while the KT remained on the same site. All players were evaluated in the three KT states with three days of exercises, and the quadriceps strength was estimated using the isokinetic dynamometer. The instructions for the three states were randomized with an irregular number generating table. To avoid any possibility of muscle fatigue from performing past isokinetic evaluations, the assessments were performed at least 7 days apart 8.

Muscle performance was evaluated with a Cypex isokinetic dynamometer. All players were given verbal directions to engage in muscle exertion and were permitted to see the screen. The assessments included the following: concentric quadriceps contraction compressions at 60°/s, eccentric quadriceps contraction at 60°/s, concentric quadriceps contraction at 180°/s, and eccentric quadriceps contraction at 180°/s. To assess the strength of the hamstring muscle, a similar test was applied by the same examiner who was blinded to the objectives and procedures of the study.

The numerical data are reported as means and standard deviations. Inferential statistics was applied through ANOVA to assess the changes in muscle strength in the three KT states, and the statistical analyses were performed utilizing SPSS version 22.0 (SPSS, Chicago, IL, USA) with a significance level of *p*<0.05.

## RESULTS

All study participants were healthy soccer players, and none of the players complained of pain or uneasiness during the assessment. The investigation results of peak torque and total quadriceps muscle strength using isokinetic evaluations are reported in Table 1. ANOVA, which assessed the measurements from the three KT conditions, found no significant differences among the players in any of the three conditions (*p*>0.05). Moreover, no significant changes were observed among the three states after the evaluations (*p*>0.05).

## DISCUSSION

The current study was designed to investigate the medical impact of KT on muscle performance in healthy young soccer players. The main outcomes of this study suggest that there were no significant changes in muscle performance, peak torque or total work measures for all players in any of the three KT conditions.

This present examination initially expected to observe lower peak torque and aggregate work in this investigation, which would have been in line with past investigations that enrolled athletes 8,9. Nevertheless, the discoveries of the present investigation were exceptionally comparable to the past discoveries.

In accordance with the results of the present investigation, Poon et al. endorsed that KT did not increase peak torque or total work or shorten the time to peak torque in healthy youthful adults. The positive outcomes of the past investigations of KT might be ascribed to placebo effects 10.

Without considering the psychological components, in past examinations, KT was successful in aiding muscle activity 2,11,12, initiating a prior event of muscle peak torque 12,13 and improving practical performance 14,15. In the present study, these previously announced findings were believed to result from placebo effects. This placebo effect is a psychological circumstance that can adjust physical states and performance based on the desire of the subjects to change their convictions, thereby prompting a noticeable positive or negative outcome 16,17.

Past research has suggested that placebo effects may have similar pain-relieving impacts to opioid or non-opioid instruments by acting on various parts of the body, such the respiratory centers and adrenal glands, to prompt a decrease in pain 18. Additionally, positive outcomes were additionally observed in patients with Parkinson's disease who were told that they were receiving Parkinson's disease medication that could enhance their motor capacity; the findings showed that the patients' desires prompted rapid neural improvements 19. Another study also demonstrated that KT has acute effects on the muscle strength and fatigue in the forearm muscles of tennis players 20. Comparable outcomes have been observed among various athletes, suggesting that the use of placebo effects can improve the athletic performance.

A review article proposed that a majority of athletes trusted that placebo effects could impact performance and confessed to having encountered placebo effects themselves 16,17. Despite the limited information available about placebo treatment approaches, the results suggest that a healthy connection between conviction and execution exists, which could influence the precision of experimental psychological research 16. Conclusively, KT has no medical impact on muscle performance in healthy youthful soccer players.

The study has some limitations: **(1)** the present examination included only youthful, healthy soccer players, which may restrict the applicability of the present discoveries to different age groups. **(2)** KT was applied with a standard amount of tape tension; however, other studies have demonstrated that applying KT at starting point with a tension of thirty-five percent can enhance muscle strength, and another study suggested that tension of fifty to seventy-five percent may achieve the same results 21. Additional studies should assess the impact of KT tension on muscle performance. **(3)** Similarly, testing for more than three days may lead to inaccurate estimations, as this study did not entirely limit the activities of the participants between assessments of the three KT conditions. **(4)** Finally, this investigation evaluated only the impact of KT application on muscle performance. Furthermore, this study did not evaluate the medical impact of KT on different issues; for example, the effect of KT on pain remains unknown.

## CONCLUSIONS

This study found that KT application has no clinical effect on muscle performance. Additionally, KT did not increase or decrease peak torque or total work in healthy young soccer players.

## ACKNOWLEDGMENTS

This project was supported by the deanship of Scientific Research at Prince Sattam Bin Abdulaziz University under research project No. 2018/03/9331.

## Figures and Tables

**Table 1:** Comparison of the peak torque and total work of quadriceps and functional activity among the three conditions.

Muscle contraction	Rate(°/s)	Condition (Mean±SD)			
		WKT	IKT	8KT	*p*-value
Peak torque (kgm)					
Concentric	60	41.7±11.4	39.2±10.7	41.4±10.5	0.713
Eccentric	60	43.7±14.3	41.8±12.4	43.2±13.1	0.641
Concentric	180	34.3±9.12	31.6±11.2	33.9±10.9	0.356
Eccentric	180	38.6±10.5	37.8±11.7	38.4±10.8	0.285
Total work (kgm)					
Concentric	60	26.8±7.6	25.7±7.4	26.5±6.8	0.394
Eccentric	60	29.2±8.7	27.8±7.6	28.8±9.1	0.542
Concentric	180	23.3±6.7	21.6±6.5	23.6±7.1	0.467
Eccentric	180	26.4±7.8	25.9±7.6	27.1±8.2	0.385

SD: standard deviation; *p*: probability; WKT: without KT; IKT: immediately after applying KT; 8KT: 8 hrs after applying KT.
